# The Blind NasoTracheal Aspiration Method Is Not a Useful Tool for Pathogen Detection of Pneumonia in Children

**DOI:** 10.1371/journal.pone.0015885

**Published:** 2010-12-29

**Authors:** Tao Zhang, Steven Black, Chuangli Hao, Yunfang Ding, Wei Ji, Rong Chen, Yuzun Lin, Juhani Eskola, Henry Shinefield, Maria Delorian Knoll, Genming Zhao

**Affiliations:** 1 Epidemiology Department, School of Public Health, Fudan University, Key Laboratory of Public Health Safety, Ministry of Education, Shanghai, China; 2 Center for Global Health, Cincinnati Children's Hospital, Cincinnati, Ohio, United States of America; 3 Suzhou University-Affiliated Children's Hospital, Suzhou, China; 4 National Institute for Health and Welfare (THL) Helsinki, Finland; 5 University of California San Francisco, San Francisco, California, United States of America; 6 Johns Hopkins Bloomberg School of Public Health, Baltimore, Maryland, United States of America; University of Stellenbosch, South Africa

## Abstract

**Background:**

Acute lower respiratory infection (ALRI) is a major cause of hospitalization for children in China, while the etiological diagnosis of ALRI remains a challenge. This study was performed to evaluate the utility of the blind Nasotracheal aspiration (NTA) in the pathogen detection in ALRI through an evaluation of the test's specificity.

**Methodology/Principal Findings:**

A hospital-based study of children ≤3 years was carried out from March 2006 through March 2007 in Suzhou University Affiliated Children's Hospital, including 379 cases with ALRI from the respiratory wards, and 394 controls receiving elective surgery. Nasopharyngeal swabs (NPS) and NTA specimens were taken on admission. *S. pneumoniae* was isolated from 10.3% of NTA samples from ALRI children, *H. influenzae* from 15.3%, and *M. catarrhalis* from 4.7%. The false positive rate—the strains from NTA in control group children—was 8.4% (95% CI: 5.8%–11.4%) for *S. pneumoniae*, 27.2% (95% CI: 22.7–31.5%) for *H. influenzae*, and 22.1% (95% CI: 18.0%–26.2%) for *M. catarrhalis.* The agreement between NPS and NTA in the control group was over 70%.

**Conclusion/Significance:**

The blind NTA test is not a useful test for etiologic diagnosis of ALRI.

## Introduction

Acute lower respiratory infection (ALRI) is a major cause of hospitalization for children in China. Pneumonia accounts for 25%–37% of the total disease burden for children admitted to medical wards each year and is the most common reason for hospitalization in those under 2 years of age [1].

Currently available diagnostic tests are inadequate to delineate causative pathogens among children with ALRI [2]–[7]. Isolation of bacterial pathogen from usually sterile body fluids such as blood or pleural fluid is highly specific, but has very low sensitivity [8]. Diagnosis based on sputum culture is controversial due to nasopharyngeal carriage of flora in healthy individuals [9]. In addition most children are unable to produce adequate sputum specimens. The lack of rapid and sensitive diagnostic tools and high morbidity associated with ALRI, and emerging antibiotic resistance strains such as *S. pneumoniae* and *H. influenzae* all support the need to improve the pathogen identification of ALRI [4], [10].

In this study we evaluated the blind Nasotracheal aspiration (NTA) test, which is frequently used as a routine diagnostic or research tool for children with pneumonia in China and in other countries in Asia [11]–[18]. We compared this NTA test with Nasopharyngeal swab (NPS) in children <3 years without ALRI to examine whether the NTA test could correctly yield a negative result in children who did not have pneumonia – i.e. to evaluate the specificity of the test. Information was also collected on children with a diagnosis of ALRI in case the specificity of the test warranted its use to evaluate the epidemiology of ALRI in this setting.

## Materials and Methods

Children aged 36 months or under and hospitalized at the Children's Hospital of Suzhou University during the period from March 2006 through March 2007 were screened for enrollment. Children with pneumonia [current pneumonia signs/symptoms including cough, tachypnea, and/or retractions, and with a pneumonic infiltrate documented on a chest X-ray and fever (≥38.0°C within 72 hours of enrollment)] were eligible for the ARLI group. Children from surgery wards without respiratory infection in the previous 14 days and without current fever or antibiotic use were eligible for the control group. In these controls, NPS and NTA samples were obtained in the first morning after their admission.

NPSs were obtained by using the BBLTM Culture SwabTM Collection and Transport System (BBL Microbiology Systems, Cockeysville, MD, USA) through the nasal cavity with the swab reaching the nasopharyngeal area. The swab remained in place for 10 seconds and was then slowly extracted. NTAs were obtained by blindly passing a suction catheter through the nose with the intent of passing it into the larynx and the trachea. The depth of penetration for the NTA catheter was set to 15–18 cm, depending on the size of child, and the NPS was inserted the length from the tip of the nose to the earlobe. Mucous was then obtained by mechanical suction into a sterile trap. NPS and NTA samples were transported to the bacteriologic laboratory without additional transport medium within 2 hours. Gram and Wright staining were performed on NTA specimens on a direct smear. Both sample types were cultured in trypticase soy broth containing 5 µg/ml gentamicin, on enriched chocolate agar plates, and on selective sheep blood agar plates containing 5 µg/ml gentamicin. Isolates were identified as *S. pneumoniae*, *Haemophilus influenzae*, *Moraxella catarrhalis*, and *Staphylococcus aureus* were identified according to the laboratory's standard operating procedure. All *S. pneumoniae* strains were kept frozen at −80°C in porous beads (MicrobankTM, Richmond Hill, ON, Canada) for further analysis.

Since there is no “gold standard” for the etiologic diagnosis of abacteremic pneumonia in children against which the NTA test could be compared, the primary focus of this study was to assess the specificity of the test. In order for the test to be useful and predict etiology, the specificity of the test would have to be high (i.e. its “false positive rate” would have to be low.) To assess this, we calculated the proportion of ALRI or control children who tested positive. We designated the presence of *S. pneumoniae* or other bacteria in the control children who had no evidence of respiratory tract infection as “false positives” and evaluated specificity accordingly. Differences in categorical measures were assessed with Pearson's χ^2^ test; *P* < 0.05 was considered significant.

The study was approved by the Institute Review Board in the School of Public Health at Fudan University which is registered with the office for human research protections and has a federal wide assurance (approval no. IRB #06-02-0040).

## Results

In total, we screened 839 hospitalized children in the Children's Hospital of Suzhou University for inclusion in the study; 66 did not meet entry criteria and were excluded. This analysis was based on data from the remaining 773 children (379 cases with ARLI and 394 controls). The mean ages were 1.26±0.79 y for ALRI children and 1.73±0.62 y for the control children. The majority was boys: 88.8% in the control group and 58.3% in the ALRI group (χ^2^ = 93.2, *P* < 0.001). Antibiotics had been administered to 86% of ALRI children within one week before admission; 80% had received one or more β-lactams, and 22% had received macrolides.

It is unexpected that the isolation rate of NPS and NTA was higher in the control group than in the ALRI group (Table 1). For instance, *S. pneumoniae* was isolated from 10.3% of NTA samples from the ALRI children, *H. influenzae* from 15.3%, and *M. catarrhalis* from 4.7%; while in the control children, *S. pneumoniae* was isolated from 8.4% of NTA samples, *H. influenzae* from 27.2%, and *M. catarrhalis* from 22.1%. There were significantly increasing number of bacteria isolated from control children than from ALRI children. In addition, the bacteria positive proportion of NTA was higher than that of NPS either in ALRI children (χ^2^_ALRI_ = 155.9, *P* < 0.001) or in control group children (χ^2^_Control_ = 66.9, *P* < 0.001).

**Table 1:** The proportion of bacterial isolates using NPS and NTA approaches in ALRI and control children.

Bacteria	ALRI (n = 379)		Control (n = 394)	
	NPS(%)	NTA(%)	NPS(%)	NTA(%)
*S. pneumoniae*	44(11.6)	39(10.3)	59(15.0)	33(8.4)
*H. influenzae*	46(12.1)	58(15.3)	63(16.0)	107(27.2)
*M. catarrhalis*	21(5.5)	18(4.7)	82(20.8)	87(22.1)
*S. epidermidis*	51(13.5)	8(2.1)	35(8.9)	13(3.3)
*S. aureus*	11(2.9)	14(3.7)	42(10.7)	65(16.5)
Number of bacterial types				
None	144(38.0)	16(4.2)	29(7.4)	6(1.5)
1	156(41.2)	172(45.4)	126(32.0)	50(12.7)
2	68(17.9)	134(35.4)	114(28.9)	141(35.8)
3+	11(2.9)	57(15.0)	125(31.7)	197(50.0)

Notes: NPS, nasopharyngeal swab; NTA, blind nasotracheal aspiration; ALRI, acute lower respiratory infection.

Since we assumed that the trachea - the NTA's target site - would be sterile in healthy control children. Therefore, positive NTA tests for control children were considered as false positives. Accordingly, the false positive rate was 8.4% (95% CI: 5.8%–11.4%) for *S. pneumoniae*, 27.2% (95% CI: 22.7–31.5%) for *H. influenzae*, and 22.1% (95% CI: 18.0%–26.2%) for *M. catarrhalis* (table 2).

**Table 2:** The false positive rate of NTA test in control group children.

Strains	NTA		Total	False positive rate (%)	*95% CI*
	+	−			
*S. pneumoniae*	33	350	383	8.4	5.8, 11.4
*H. influenzae*	107	287	394	27.2	22.7, 31.5
*M. catarrhali*	87	307	394	22.1	18.0, 26.2

Notes: NPS, nasopharyngeal swab; NTA, blind nasotracheal aspiration; ALRI, acute lower respiratory infection; CI, confidential interval.

If the NTA test results were closely related to nasopharyngeal carriage, they could yield high agreement and kappa value between NTA and NPS. In this study, the agreements between NTA and NPS were over 70% in the control children, and above 90% in ALRI children. In addition, the kappa value varied from 0.241 to 0.751 in the control children group and above 0.5 in ALRI children (table 3). Thus, the NTA test results were closely related to nasopharyngeal carriage and it's likely contaminated by flora from upper pharynx in children without pneumonia as well as those with ALRI.

**Table 3:** The agreement of bacterial cultures between NPS and NTA.

	ALRI		Control	
NTA vs NPS	Agreement(95%*CI)*	kappa	Agreement(95%*CI)*	kappa
*S. pneumoniae*	0.929(0.903–0.955)	0.635	0.864(0.830–0.899)	0.365
*H. influenzae*	0.889(0.858–0.921)	0.533	0.741(0.698–0.784)	0.241
*M. catarrhalis*	0.966(0.947–0.984)	0.649	0.916(0.889–0.944)	0.751

NPS, nasopharyngeal swab; NTA, blind nasotracheal aspiration; ALRI, acute lower respiratory infection; CI, confidential interval.

## Discussion

Our study showed a high rate of positive bacterial cultures from the blind NTA test in the control group. It is possible that the quality of the NTA results was poor because the utility of the test depends on the ability to blindly pass a catheter into the trachea. If it is not possible to blindly pass a catheter into the trachea using the method described, we would expect contamination of the specimens by the flora of the oropharynx as we observed in this study. In our study, 22 of the 773 children (2.8%) have 10 or more squamous cells per high-power field in their NTA's direct smear, consistent with results from other studies that would indicate that the specimens were of good quality [19], [20]. However, this criterion has only been used to assess sputum contamination in adults and may not be valid in children.

Although the NTA test is frequently used in China and elsewhere in Asia for children with ALRI, our results demonstrated that it is not sufficiently specific for determining the etiology of ALRI. Firstly, contrary to our hypothesis, there was over 20% healthy control children had the *H. influenzae* and *M. catarrhalis* isolates from NTA specimens. Secondly, we observed that over 98% children without any respiratory infection had positive cultures and 85% children had two or more strains in the NTA specimens. Thirdly, the agreements and the kappa value between NPS and NTA demonstrate that the NTA and NPS were highly related both in ALRI or control group children. In order to avoid observational bias from laboratory in our study, the laboratory technicians were blinded and were unable to determine the subjects' group and to link any NPS with corresponding NTA specimen. Thus, it is very likely that many of the samples were indeed contaminated by flora from upper pharynx, and in most cases the NTA measures only carriage.

There are some potential concerns for the study. Firstly, the age and sex distribution between the two groups differed significantly. Secondly, there was some loss of patients after screening. Thirdly, the extensive antibiotic use in ALRI children could have suppressed the flora in the nasopharynx. Although such high levels of pretreatment with antibiotics would be problematic if our primary goal was to assess the epidemiology of pneumonia in these children, for our primary objective of assessing the specificity of the test, this antibiotic pretreatment may have reduced the carriage rates overall but should not have had an effect on our assessment of specificity since our conclusions are mainly based on the results from control children.

In summary, although it is frequently used for diagnostic purposes in Asia, the blind NTA test is likely contaminated by flora from upper pharynx, and not sufficiently specific to warrant its use the etiologic diagnosis of pneumonia in clinical or epidemiologic studies.
